# Entropy generation analysis and thermal synergy efficiency in the T-shaped micro-kenics

**DOI:** 10.1016/j.heliyon.2024.e32233

**Published:** 2024-06-03

**Authors:** Abdelkader Mahammedi, Naas Toufik Tayeb, Jin-Hyuk Kim, Shakhawat Hossain

**Affiliations:** a Department of Mechanical Engineering, University of Djelfa, Djelfa, 17000, Algeria; b Renewable Energy Systems Applications Laboratory (LASER), Gas Turbine Joint Research Team, Ziane Achour University, Djelfa, 17000, Algeria; c Carbon Neutral Technology R&D Department, Korea Institute of Industrial Technology, Cheonan, 31056, South Korea; d Convergence Manufacturing System Engineering (Green Process and Energy System Engineering), University of Science & Technology, Daejeon, 34113, South Korea; e Department of Industrial and Production Engineering, Jashore University of Science and Technology, Jessore, 7408, Bangladesh

## Abstract

In this work, three different twist angles of a micro helical insert in a T-shaped are studied numerically in order to evaluate the laminar steady flow behavior of Newtonian fluid in chaotic geometry. In the geometries under consideration, thermal mixing behavior is carried out using fluids having two distinct input temperatures. Under the influence of chaotic advection and low rates of Reynolds number, the second law of thermodynamics is controlled in terms of the entropy generation caused by hydrodynamic and thermal processes. The governing equations are numerically solved using the CFD Fluent code. Thus, the micromixer's configuration demonstrated a very significant improvement in mixing degree while minimizing friction and thermal irreversibilities. The synergy coefficient, which depicts the link between velocity and heat transfer in angle form, is analyzed and the results are provided.

## Highlights

• Novel numerical work of Newtonian fluid mixing has been performed using efficient chaotic kenics mixer.
• High mixing index has been obtained using for the kenics micromixer, which exceeds 0.97 for all values of Reynolds number.
• The T-micromixer has shown the lower pressure drops (Po/MI) compared to other proposed geometries.
• Very low rates of mixing energy cost (MEC) have been obtained using kenics micromixer.

## 1 Introduction

Thermodynamic studies of energy transformation efficiency, especially those utilizing the second law of thermodynamics, are a focus of scientific inquiry, particularly given the interest in the efficient use of all energy sources.

Mixing heated fluids in modern micromixers is a critical challenge in multiple transport processes of constructing microscopic devices with significant value in thermal chemical engineering. Over the years, several studieshave recognized the heat thermal problem and provided ways to improve the thermal capacities of mixing fluids while lowering pressure drops. However, because of the miniature size of these concepts, which are typically of smooth microscopic order, it might be hard of speeding up the molecular diffusion mechanism. Despite inadequateflow mixing, an improvement in hydrodynamic and heat flux is limited. Numerous research teams improved the mixing hydrodynamic and heat transfer rate using a variety of advanced geometries [[1], [2], [3], [4]].

The chaotic advection approach is used for being one of the best potential passive mixing techniques to improve flow mixing. The T-Shape mixer, whose 3Din laminar steady flow is one of the probable chaotic geometries that may offera reasonable strategy to improving the hydrodynamic and fluid kinematics performances. Some of the researchers who used such geometry in their investigations [[5], [6], [7], [8], [9], [10]], they empirically and numerically proposed numerous patterns for implementing chaotic advection to passively boost fluid mixing. According to their findings, the mixing rate of the T-shaped mixer grows as the Reynolds number increases.

Many research teams used numerically complex geometries to improve mixing heat transfer and minimize pressure drop in related to entropy generation [[11], [12], [13], [14], [15]] the entropy generation rate estimates how much energy is lost in a system due to irreversibility. Thegeometry of a channel can have a large impact on the quantity of entropy created.

With the degree of mixing depending on the Reynolds number, Mariotti et al. [9] found that stratification can have a significant impact on reaction yield and mixing. They looked into the effects of stratification on mixing and chemical reactions in a T-shaped microreactors, discussed the practical limitations of using micro-reactors, and emphasized the significance of optimizing mixing in liquid reactions. Using CDF software, Shakhawat et al. [16,17] demonstrated numerically the mixing of dye-water and water within an OH-shaped geometry. Then they suggested a unique complex geometry termed the SAR micromixer, using CFD code. In order to demonstrate the impact of low Reynolds number on the improvement of mixing performance, Kim et al. [18] collected experimental data for the (SLM) serpentine micromixer, a unique chaotic micromixer. These conclusions were established, however, by a quick mixing employing Split and Recombine of flow in 3D micromixer for low Reynolds number. The fluid mass fraction was utilized to calculate the mixing efficiency inside the micromixers, after Xia et al. [19] confirmed the results of the SAR micromixer. The experiment and their findings were in good agreement. For Reynolds numbers in the range of 0.2 to 120the serpentine micromixer demonstrated no less than 96 % mixing. They noticed that the two-layer serpentine micromixer displayed less pressure losses than that attained with comparable geometry at Reynolds numbers greater than 10. Anelevatedcapacity of aqueous solutions with enhanced mixing due to vortical structures createdwithin the micromixers are provided by Kockmann et al. [20,21] they presented the construction and design, and mixing parameters of various mixer models, includingthree T-mixer variations while another tangential mixer.

In order to ascertain the impact of asymmetrical flow conditions at the inlets as well as the production of secondary flows and vortexat the junction on mixing, Wong et al. [22] employed numerical simulations and experiments in micro T-mixers. For better fluid mixing for Re > 20, Matsunaga and Nichino [23] included two anti-symmetric barriers in the input tubes close to the T-mixer's junction area. These barriers create angular momentum in the flow and provide a swirling flow contour.

Bothe et al. [24] investigated mixing capabilities in T-formed micromixer and concluded that only the engulfment flow with input stream intertwinement results good mixing by rolling up the initial planer contact surface. They used a trio flow regimes.

Gobby et al. [25] quantitatively examined the T-shape microfluidic mixer in 2-D to determine the mixing properties for gas flow as well as the effect of flow rate and various design parameters on the needed mixing length. A passive micromixer was developed by Yang et al. [26] in which surface tension-driven counterflow of two fluids in a chamber creates a huge 3-D flow vortex that enhances mixing. Chen et al. [[27], [28], [29]] developed a T-microchannel with obstacles and special zigzag micromixer to improve mixing. The secondary flows in the arrangement can aid to improve chaotic advection. They discovered that mixing performance exceeds 93 % over a wide range of Reynolds numbers starting with Re = 5. The barriers may be utilized in systems for chemical engineering.

The second-law aspect of heat transmission was described through several instances of fundamental forced convection difficulties by Bejan [[30], [31], [32]], among the earliest authors who examined the optimum mechanism approach of second-law in order toexplore the entropy generation method (EMG).

Naas et al. [33] reported that the secondary flow rate of the TLCCM shape was greater than that of the C-shaped and serpentine designs in their examination of the entropy generation features and thermal mixing capabilities of a shear-thinning flow utilizing TLCCM. Haddad et al. [34] used numerical methods to evaluate the entropy generation in parallel plate microchannel. They discovered that as the Knudsen number rises, so does the entropy generation within the microchannel, which rises as the Prandtl, nondimensional variation in temperature and Reynolds rises. Furthermore, as Knudsen number increases over a wide range of flow controlling parameters, the contribution of viscous dissipation to total entropy generation increases. Selimefendigil et al. [35] explored theMHD mixed convection and entropy generation in a lid-driven cavity filled with nanofluid. They discovered that the addition of nanoparticles, particularly for the bottom triangular domain, is an efficient technique to improve heat transfer when the heat transfer is strong and convection is not slowed by lowering the Hartmann number. Entropy generation in an evenly heated microchannel heat sink was examined by Abbassi et al. [36]. They employed a porous media model based on the additional Darcy equation for fluid flow and the two-equation approach to heat transfer to solve the forced convection problem across the microchannel analytically. They discovered an assumed value of porosity where the rate of entropy creation is at its lowest level. In a cavity filled with nanofluid exposed to a regular magnetic field, Chamkha et al. [37] examined the formation of entropy and spontaneous convection. They discovered that both natural convection and the rate of entropy production can be decreased by the applied magnetic field.

Erbay et al. [38] used numerical methods to explore the entropy formation in microchannels caused by forced convection in transient laminar regime at the entry area amid two parallel plates. They discovered that at the highest Reynolds numbers, entropy formation is at its highest value. The second law analysis has been used by Avci et al. [39] for two distinct microgeometries: microtubes and microducts. They examined fully developed flow in both thermal andhydrodynamic aspect.

According to Sadeghi et al. [40], who used the second law of thermodynamics to examine the thermal and hydrodynamic fully developed flow of gas in laminar regime with asymmetrically heated walls within annulus microchannels, they discovered that entropy formation rises with escalating values of Brinkman number and decreases with increasing Knudsen number. It also rises as the ratio of Brinkman number to dimensionless temperature difference increases.

ArashJafari et al. [41] discussed the usage of EGM in micro channel heat sinks. They investigated the rate of entropy as a function of Reynolds numbers, as well as the effects of pumping power, channel number, and diameter. In the study of Shu-Min Tu et al. [42] the first and second laws of thermodynamics are used to optimize heat transmission in X-shaped microchannels. They observed that lowering entropy generation often improves mixing. Gillispie et al. [43] explain the derivation of an analytical connection for the mixing efficiency and entropy value as a function of the Reynolds number. The results enable an evaluation of chaotic advection significance and the mixing factor in micromixers.

Yilmaz et al. [44] discussed the importance of systematic heat exchanger design utilizing second law analysis. A performance evaluation criterion based on second law analysis was established for heat exchangers. They revealed that several criteria of the second law performance, such as employing exergy or entropy as an evaluation parameter have relation to one another, and that consideration should be given to both the constraints and the characteristics when designing heat exchangers.

The exergy approach differs from the entropy generation lowering technique in that it only employs the first and second laws, as well as environmental properties. EGM characteristics, on the other hand, include system modeling, the evolutionof $s_{gen}^{\prime \prime \prime }$ as a parameterof modelfunctions, and the capacity to slow down the pace of entropy creation. Because of the irreversibilities, the first law is insufficient to determine the thermal performance of any thermal system. The system consumes exergy due to inefficiency in its capacity to transfer available energy. Consideringthe flow in heat exchanger, which employs both cold andhot fluids [45].

The primary goal of this article is to examine how the geometry configuration affects the mass and thermal mixing performance and the rate of entropy generation while accounting for heat transfer, flow friction, synergy, and using 3D Navier-Stokes equations in the T-shaped micro-channel within hot and cold fluid over a Reynolds number interval of 1–60. Micro-helical implanted in T-microchannels is taken into consideration from three different twisted angles. The study reveals the energy potential of microfluidics, which can be used to improve the mixing performance and reduce energy consumption. It also has the potential to accelerate chemical reactions, which is useful for energy generation processes, and offers innovative insights into sustainable energy solutions.

### 1.1 Governing equations

Steady conservation equations of incompressible and steady flows are solved numerically by using a CFD code Ansys fluent 16.0, which uses a control volume technique to discretize the conservation equations. These equations of three dimensional are continuity, momentum, and species mass fraction convection diffusion can be expressed in these terms [46,47]:

(1) ∇U=0

(2) $$\rho U∙\nabla U=-\nabla P+\mu \nabla^{2}U$$

(3) $$U∙\nabla C=D\nabla^{2}C$$

The expression for the energy equation is [48]:

(4) $$\rho cV∙\nabla T=k\nabla^{2}T$$

In these equations, the variables *U* denote the velocity, ρ for fluid density, *P* for static pressure, μ for viscosity, *C* for concentration, and *D* for diffusion coefficient. Where c, T, and k stand for the fluids' heat capacity, temperature, and thermal conductivity respectively.

### 1.2 Mixing index

To compare the performance of the micromixers, mixing index is formulated as below [48,49]:

(5) $$MI=1-\frac{\sigma }{\sigma_{0}}$$

Where σ is the mass fraction's standard deviation, the definition is:

(6) $$\sigma^{2}=\frac{1}{N}\sum_{i=1}^{N}{\left(C_{i}-\bar{C\;}\right)}^{2}$$

With *N* denoting the number of sampling points throughouttransversal section, $C_{i}$ is the mass fraction atinspecting point *i*, the ideal mixing mass fraction is $\bar{C}$, and the standard deviation (SD) at the inlet section is $\sigma_{0}$.

The following formula determines the highest (SD) for the data range:

(7) $$\sigma_{0}^{2}=\bar{C}\left(1-\bar{C}\right)$$

To quantify the thermal mixing we use the TMI (thermal mixing index) of the hot and cold fluids which is calculated as follows:

(8) $$TMI=1-\frac{\sqrt{\frac{1}{n}\sum_{i=1}^{n}\;{\left(T_{i}-\bar{T}\right)}^{2}}}{\sigma_{0}}$$

Where $T_{i}$ represents the temperature on node *i,* and $\bar{\;T}$ is the average temperature at the selection plane.

### 1.3 Mixing energy cost (MEC)

A higher mixing index corresponds to higher flow rates and higher energy usage. It must be estimated to balance the cost of mixing with its value efficiency terms of input power (the energy necessary to drive the fluid down the channel) andis described in this way [50]:

(9) $$MEC=\frac{\Delta P\times Q}{MI}$$

Where Q is the volume flow rate (m^3^/s) and ΔP is the pressure drop (Pa).

### 1.4 Entropy generation analysis

The irreversibility of the local entropy production owing to heat transfer (*S*_*T*_′′′) and the fluid friction irreversibility (*S*_*P*_′′′) of three-dimensional Newtonian flow can be determined by utilizing the temperatures and velocity distribution of the flow field as [51]:

(10) $$s_{T}^{\prime \prime \prime }=\frac{\lambda }{T^{2}}\left[{\left(\frac{\partial T}{\partial x}\right)}^{2}+{\left(\frac{\partial T}{\partial y}\right)}^{2}+{\left(\frac{\partial T}{\partial z}\right)}^{2}\right]$$

(11) $$s_{P}^{\prime \prime \prime }=\frac{\mu }{T}\left[2\left({\left(\frac{\partial u}{\partial x}\right)}^{2}+{\left(\frac{\partial v}{\partial y}\right)}^{2}+{\left(\frac{\partial w}{\partial z}\right)}^{2}\right)+{\left(\frac{\partial u}{\partial y}+\frac{\partial v}{\partial x}\right)}^{2}+{\left(\frac{\partial u}{\partial z}+\frac{\partial w}{\partial x}\right)}^{2}+{\left(\frac{\partial v}{\partial z}+\frac{\partial w}{\partial y}\right)}^{2}\right]$$

The sum of the entropy generation in the fluid flow, which is meant to provide a thorough description of fluid homogenization, is able to be computed as follows:

$$s_{gen}^{\prime \prime \prime }=s_{T}^{\prime \prime \prime }+s_{P}^{\prime \prime \prime }$$

Bejan number is used to calculate the ratio between thermal and total losses [52]:

(12) $$Be=\frac{S_{T}^{\prime \prime \prime }}{S_{gen}^{\prime \prime \prime }}$$

## 2 Geometry and grid

### 2.1 Geometries description

**Fig. 1:**
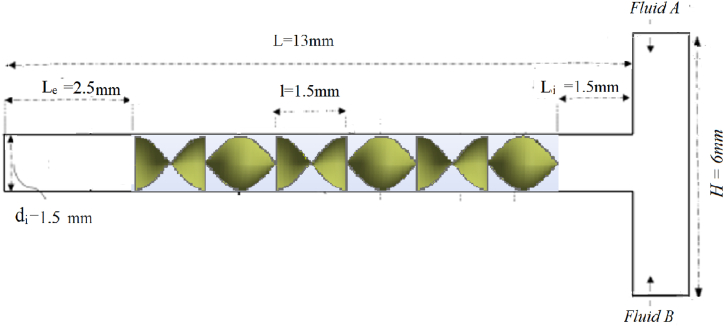
Schematic diagram of the T-micro kenics with θ = *90°*.

The geometry of the model simulated is inspired from the Kenics static mixer (KM). It comprises of a T-shaped component with a 1.5 mm diameter, and a tubelength *L* = 13 mm, using 6 helical elements. Every element has a thickness of *t* = 0.05 mm and length *l*_i_ = 1.5 mm. At a distance of *l* = 2.5 mm from the tube exit, the last helical blade element is located (Fig. 1).

**Fig. 2:**
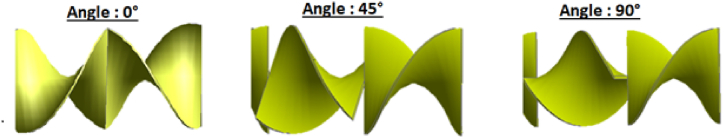
Schematic illustration of the helical element inserts with connection angles of 0°, 45° and 90°.

Theseries of 6 repetitive pieces of micro helical are assembled at a choice of connection angles and consisting of the mixing elements embedded in the pipe. Theeffect of blade twist (θ) is examined and three geometrical patterns are investigated for thispurpose, which are: θ = 0°, 45°and 90°(fig.2).

**Fig. 3:**
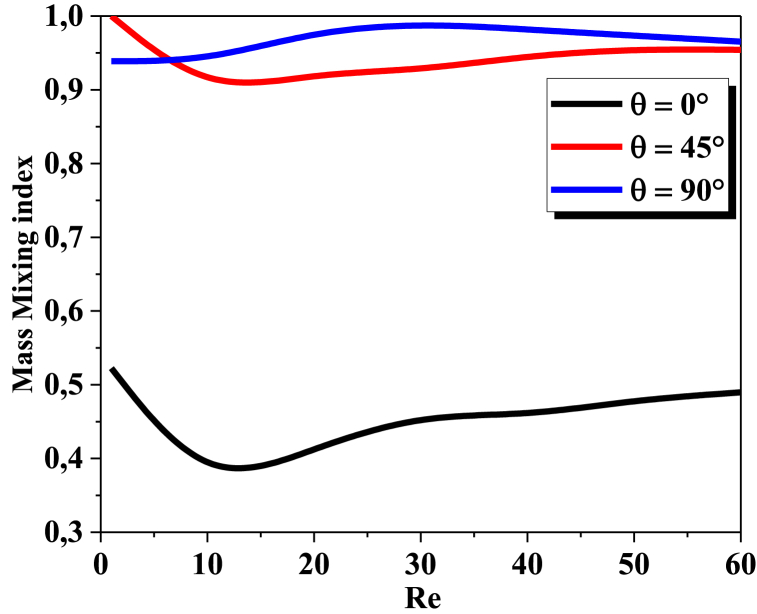
Mass mixing index vs Re

### 2.2 Boundary conditions

The boundary condition of T-microchannel wall is no-slip condition, and atmospheric pressure at the outlet, and all walls are considered adiabatic. Equal flow rates were considered at both inlets. In case of thermal mixture: hot and cold liquid temperatures are separately 330 and 300 K. The governing equation (1), (2), (3) were solved using the commercial code ANSYS® Fluent, which employs the finite volume approach.

### 2.3 Validation of numerical code

In order to verify the accuracy of our numerical code, the current simulation results were validated with the Elliptic Barriers from Lee et al. [8] at Re = 8 with 5, and 8 barriersas displayedin Table 1. It was observed thatthe error between our simulation results and these fromLeeet al.was substantially less than 1 %.

**Table 1:** Mixing efficiency comparison with Lee et al. [8].

Re = 8	Mixing efficiency %	
Number of Barriers	5br	8br
Lee et al.	60.80	82.07
Present simul.	60.72	81,38
error	0.13 %	0.84 %

## 3 Results and discussion

The diagram of Mass mixing index as a function of Reynolds numbers is a graph that shows how the mixing index changes with increasing Reynolds numbers. The mixing index is a measure of how well two fluids mix together, and it was computedat the outlet of the T-micromixer with Reynolds number from 0.1 to 60, for a three twisty angles (0°, 45°, and 90°). In Fig. 3 for all Reynolds numbers, in the helical with twisty angles 45°and 90°, the mixing index typically increases as well, indicating that the homogenization of the two fluids are becoming more thoroughly reachedand show much higher mixing performance than the helical with θ = 0°.The geometry with θ=45° has considerably higher mixing index values due to the diffusion regime at very low Reynolds. In moderateReynolds the mixing of T-helical with 90° increases to reach 0.99 % in Re between 25 and 35, due to the highly chaotic advection impact.

**Fig. 4:**
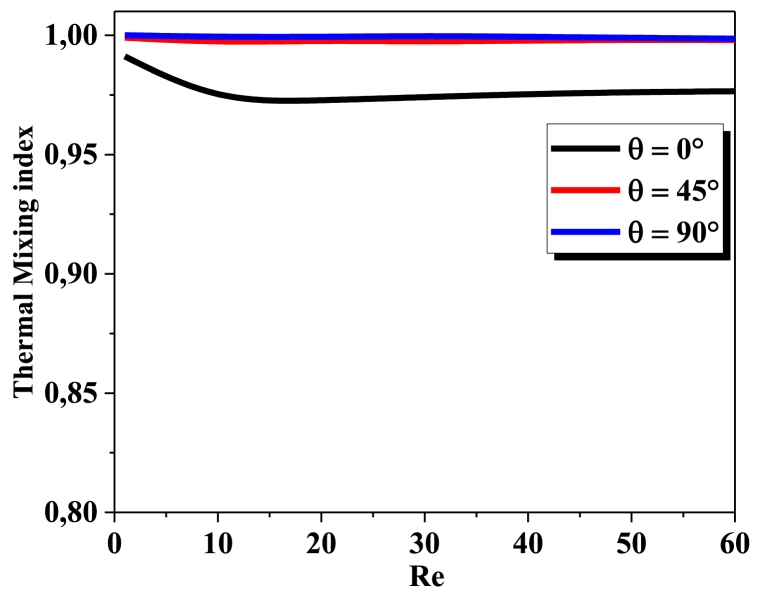
Thermal mixing index vs Re

The thermal mixing index is a measure of the degree of thermal mixing in a fluid flow. It is identified as the ratio of the temperature variance to the mean temperature. Fig. 4, depicts the deviation of TMI (thermal mixing index) at the outlet of the T-microhelical as a function of the Reynolds number. The geometric of all twisted micro helical improves the thermal mixing capabilities, and the best TMI is obtained with θ = 90°, 45°, and TMI approach to 1 (full mixing).

**Fig. 5:**
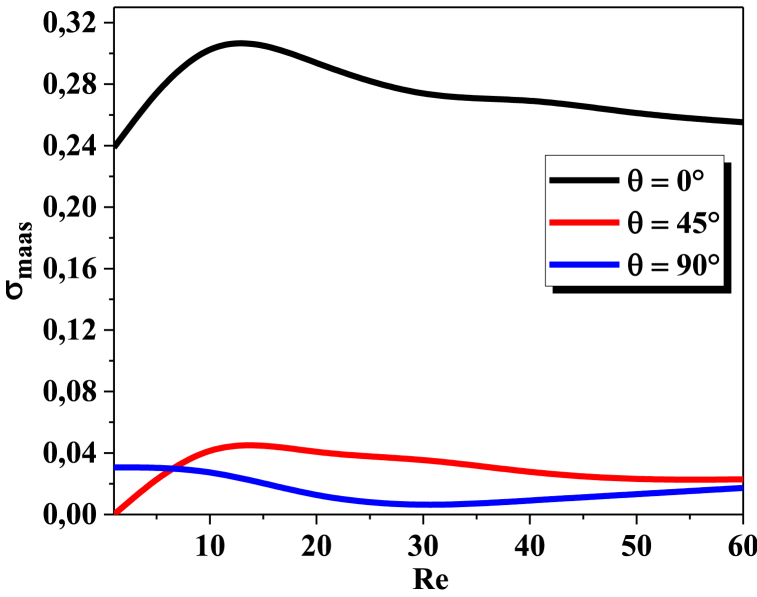
Mass Standard deviation vs Re

The graph of mass standard deviation as a function of Reynolds numbers is typically a bell-shaped curve. As the Reynolds number increases, the mass standard deviation initially increases, reaches a maximum, and then decreases again. The maximum value of the mass standard deviation occurs at the transition regime between diffusion and advection. The minimum value of the mass standard deviation occurs with θ = 90°, (fig.5).

**Fig. 6:**
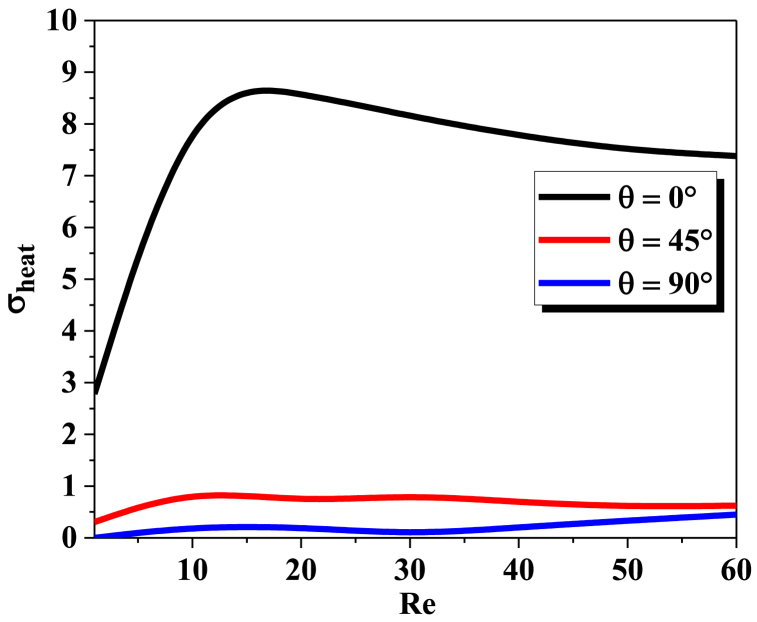
Heat Standard deviation vs. Re

The graph of heat standard deviation as a function of Reynolds numbers was shown in Fig. 6. At low Reynolds numbers, the heat standard deviation is low, but as the Reynolds number increases, the heat standard deviation increases until it reaches a peak. After this peak, the heat standard deviation decreases again as the Reynolds number continues to increase, the elevated value was obtained with θ = 0° due to low advection.

The mixing efficiency to pressure drop ratio can be utilized for calculating mixing cost. In general, a higher mixing index is associated with higher flow rates with higher energy consumption, therefore, the mixing cost required to characterize mixing performance. Generally, as the Reynolds number increases, so does the energy cost associated with mixing, additionally, the shape and size of the mixing vessel can also affect the energy cost associated with mixing.

**Fig. 7:**
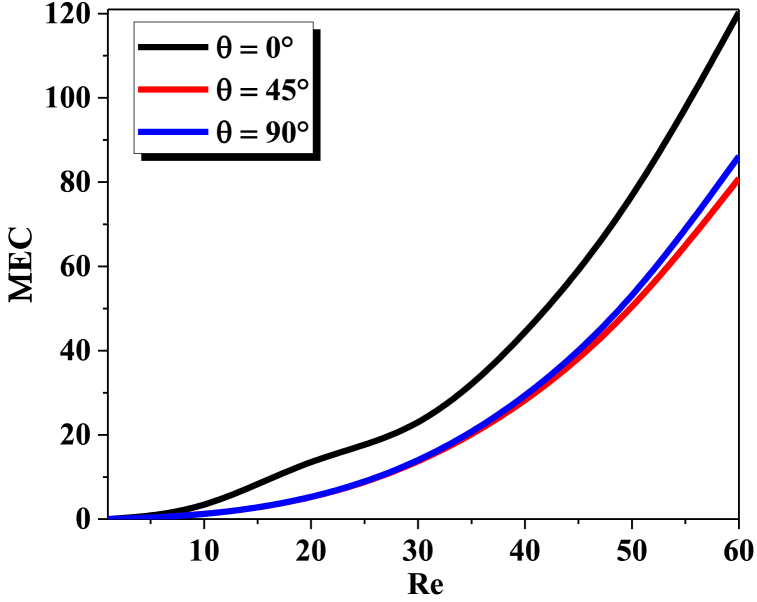
Mixing energy Costsvs. Re

Fig. 7 shows an evaluation of the cost of mixingenergy (MEC) in terms of Reynolds numbers with various twisted angles for different Reynolds numbers. We Note that the more Reynolds increases, the cost of mixing energy increases, and this is due to Pumping liquid into T-shaped as it should be noted that the lowest value of (θ) take the highest cost values.

**Fig. 8:**
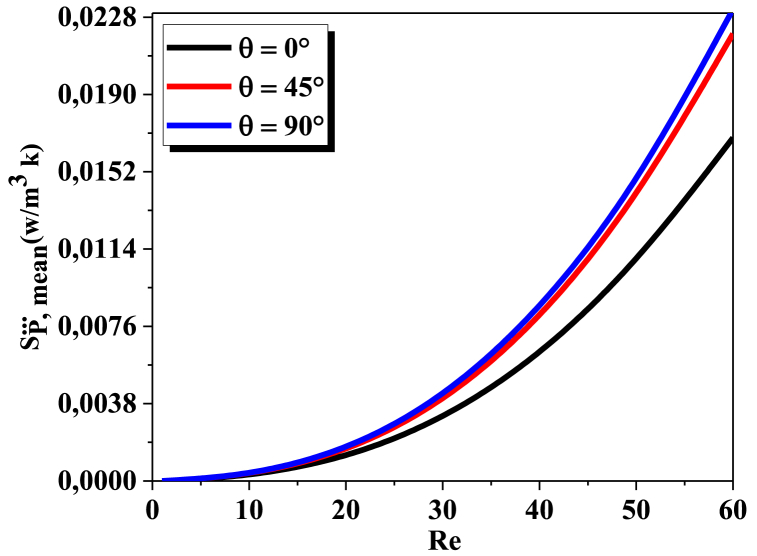
Comparison of local frictional entropy generation for Kenics micromixer, with different θ and various Re.

The local frictional entropy generation will depend on the specific geometry of the micromixer, which is relatively low in laminar flow. As the value of Reynolds increases, the inertial force gets more prominent, leading to create more entropy generation. Furthermore, as illustrated inFig. 8 entropy generated by fluid friction is little lower in θ=0° than in other geometries. As shown in Fig. 8, for all Reynolds number values the frictional entropy generation increases as twisted angle increases and leads to increase of irreversibility due to higher related pressure drop. However, the effects of the variation of twisted angle between (45° and 90) are relatively insignificant.

**Fig. 9:**
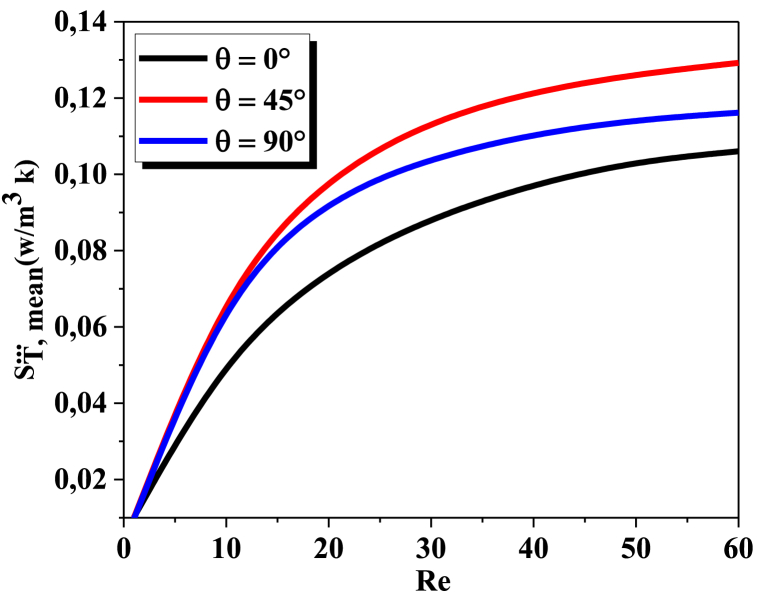
Heat transfer entropy generation for the micromixer with different θ and varying Re

The heat transfer entropy generation for a micromixer with various Reynolds numbers and different helical angles can vary significantly. As seen in fig.9, as the value of Reynolds rises, the heat transfer entropy generation augments. This is for the reason that higher Reynolds numbers indicate higher flow velocities, which can lead to increased turbulence and mixing of the fluids, resulting in greater heat transfer entropy generation. Additionally, increasing the helical angle of the micromixer can also lead to an increase in heat transfer entropy generation. This is because a larger helical angle creates more turbulence and mixing of the fluids, leading to greater heat transfer entropy generation. The largest entropy generation takes place for θ=45° , which the thermal entropy generation irreversibility is greater than that in the other configurations. As seen in Fig. 8, Fig. 9the entropy formation resulting fromheat transfer irreversibility is substantially more than that generatedby fluid friction irreversibility.

**Fig. 10:**
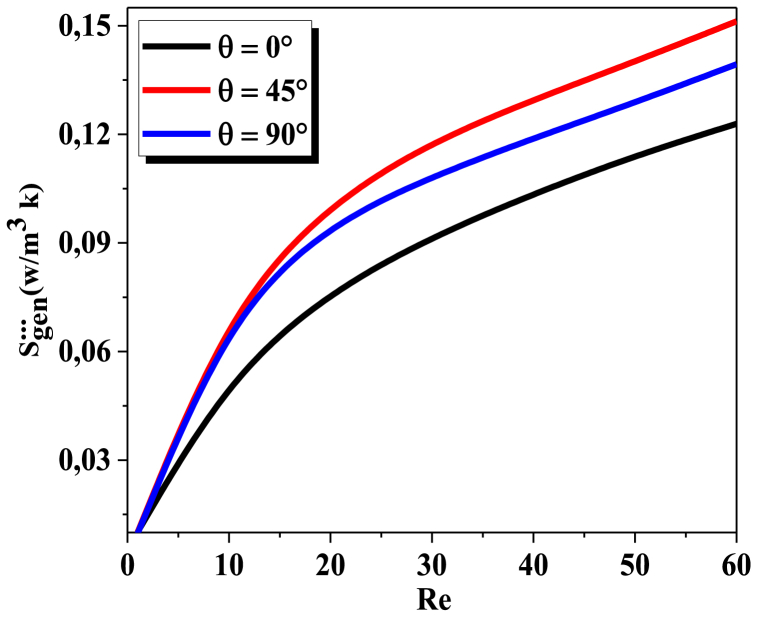
Comparison of global entropy generation for Kenics micromixer, with different θ andvarious Re.

Fig. 10 shows the variations of the global entropy generation that corresponds to the total of the rates of entropy generation, due to frictional entropy generation SP and entropy generation rate as a result of heat transfer with different Re for three values of θ, for all geometries, as Re boosts, so does the generation of heat transfer entropy. The cause of this behavior is that the temperature gradients within the flow augment. Furthermore, the highest entropy generation appears in a case of = 45°.The outcomes demonstrate that θ = 0° can boost heat performance withrespectto heat flow irreversibility by reducing fluid flow resistance.

**Fig. 11:**
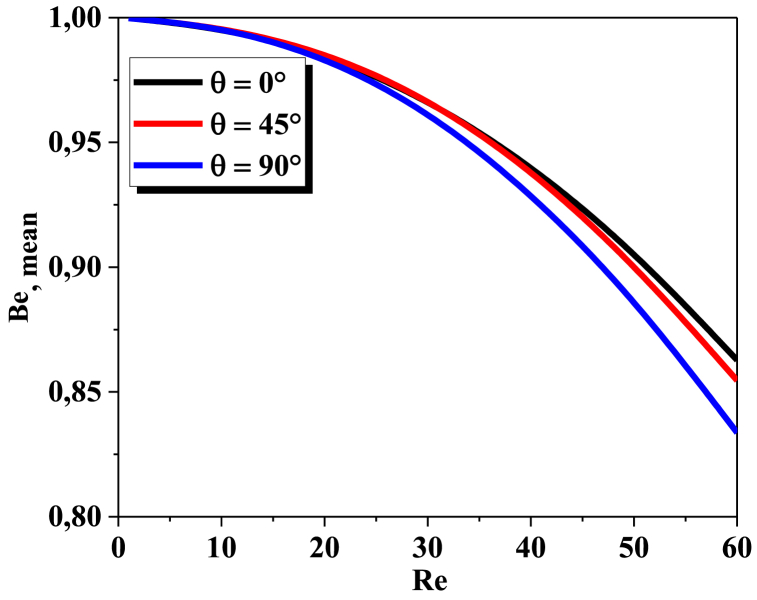
Mean Bejan number vs Re for angles ranging from *0°* to *90°*.

The Bejan number is defined as the ratio of thermal irreversibility to total irreversibility, which includes both thermal and frictional irreversibility. The Bejan number can vary depending on several factors, including geometrical forms and Reynolds number. Fig. 11 shows Bejan number versus Reynolds numbers for three values of helical angles number at θ = 0°, 45° and θ = 90°. The Bejan number for all configurations reaches unity when Reynolds number is near to zero and declines to a smallest value, the Bejan number for θ = 0° grows with smaller gradient than the two other helices. As a result, at low Reynolds numbers, we can see that the Bejan number drops as the angle value increases. That indicates that the twisted helical configuration with = 90° has the best results in terms of fluid dynamics and thermal mixing.

The probability density function (PDF) of synergy at the outlet cross section for different geometriesat different Reynolds numbers can be determined by solving the Navier-Stokes equations. The PDF of synergy is a measure of the probability that a given flow field will have a certain amount of synergy at the outlet cross section. In general, the PDF is dependent on the geometry and Reynolds number of the flow field. The Principle of Field Synergy requires that heat transfer by convection is a functionof the scalar product between the speed and the temperature gradientand therefore alsoof the direction of these two vectors. It can then be a privileged tool for qualifying andexplaining the local increase in heat transfer processes.

**Fig. 12:**
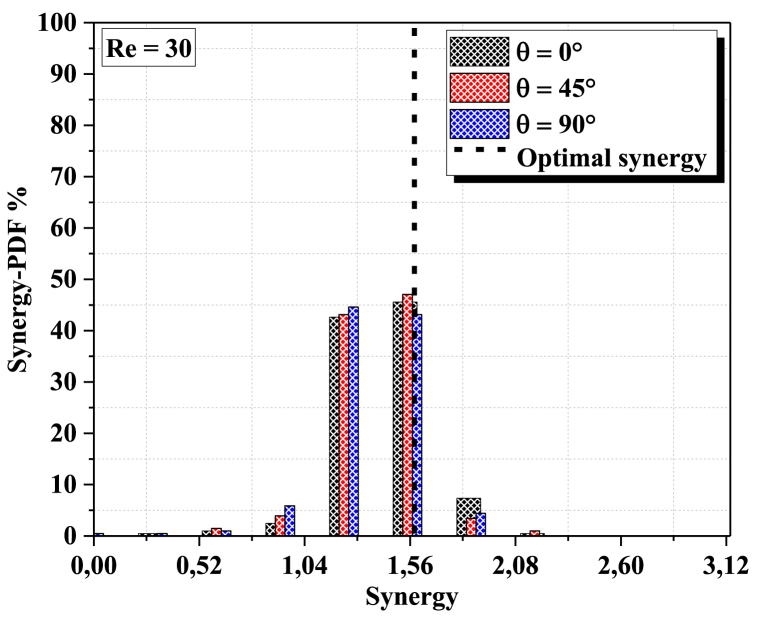

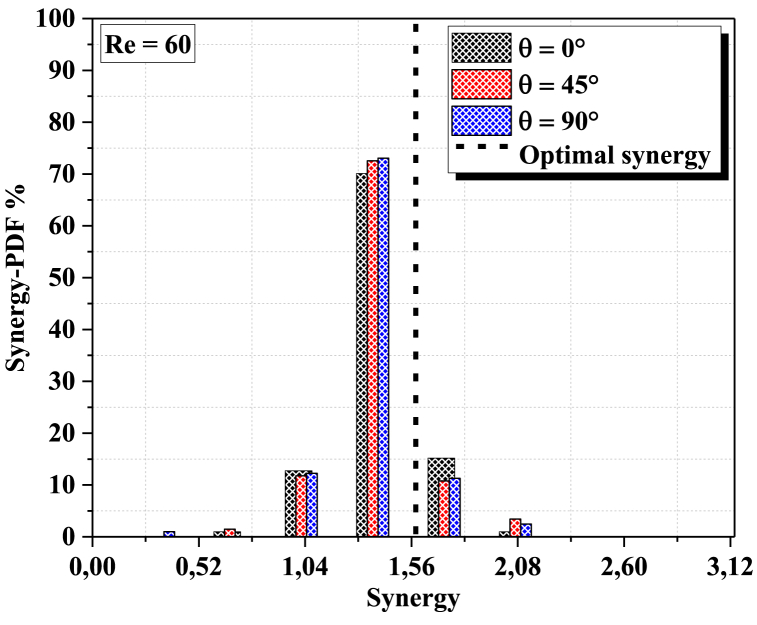
(a): probability density function of synergy at the outlet cross-section for different angles (0°, 45° and 90°) with Re = 30. Fig. 12(b)probability density function of synergy at the outlet cross-section for different angles (0°, 45° and 90°) with Re = 60.

Fig. 12a, b shows the PDF probability density function (%) of synergy at the output section, in the considered geometries, for two values of the Reynolds numbers (Re = 30 and Re = 60), it is clearly shown that the synergy in the output section is dispersed into only two parts This is an indication of thethermal mixing quality of our fine micro mixer. For Re = 60 thechaotic behavior has a strong effect on the fluid homogenization for all configurations. So, the PDF at the outlet plane of the configuration twisted with θ=90° is concentrated in a narrowof 1.56 which is corresponding to the preferred thermalmixing fluid.

**Fig. 13:**
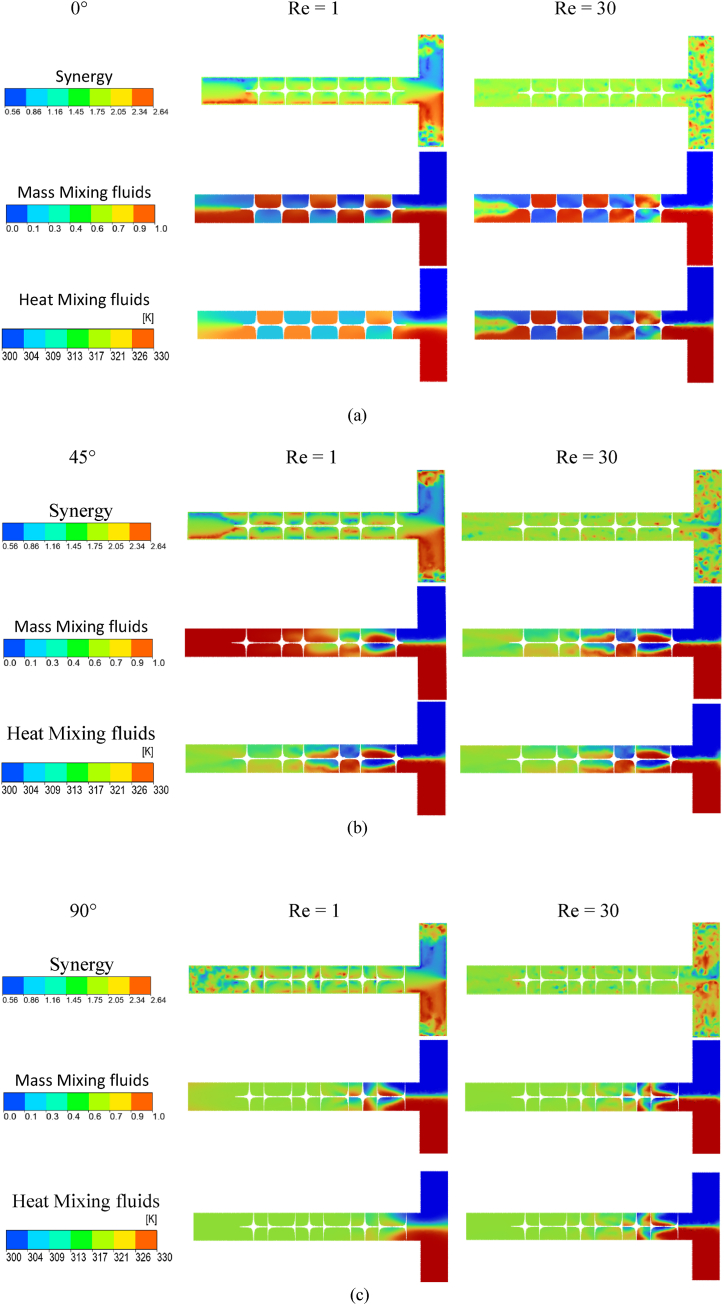
Mass and thermal mixing with synergy contours for Re = 1 and Re = 30 and (a) θ=0°,(b) θ=45°,(c) θ=90°.

Fig. 13 shows the profiles of the Synergy, Heat Mixing fluids and Mass Mixing fluids between the three geometric domains of different twisted angles θ. In order to observe the effect of secondary flux generated by eddies on heat transfer. It can be seen from the figures that synergy is important in the eddy regions of the flow. From the obtained results we found that our mixer was carried out in order to evaluate the thermal performance by calculating the synergy angle between the velocity and temperature domains. The field synergy principle has been used locally to describe convective heat exchanges.

Regarding the chaotic geometry, the thermal mixing distribution of twisted tape with θ=0° is completely different from Re = 1 to Re = 30, to that obtained by the two other geometries. As the fluid passes through the geometry, the fluids are well mixed and tend to be homogenized under the effect of the chaotic behavior of the flow, and in diffusion regime with Re = 1.

We can see that the twisted angles have a high effect on the mixing process, and the geometry within θ=90° get a high homogenization than other geometries.

The variations in thermal performance are evaluated and analyzed on the basis of changes of local velocity fields and temperature gradients locally employing the principle of field synergy.

The synergy cartography differs only slightly between the three geometries. Increasing the Reynolds number slightly improves the synergy between the velocity and temperature gradient (called synergy angle), which is the most fundamental reason why the longitudinal vortex generator can improve heat transfer. It can be see that the localized increase in synergyand heat transfer is obtained in all geometries.

## 4 Conclusion

In this study, we characterized the thermal mixing in T-micro kenics for three twisted angles, the selected Reynolds number is ranging from 1 to 60. The behavior of the heated fluid and flow kinematics, presented in terms of mass mixing and heated mixing performance, entropy generation and probability density function of synergy and Mean Bejan number is investigated in order to understand the flow pattern. Considered parameters show the effect of the angle of the twisted helical geometry.

The T-microchannel has the potential to improve mixing level while increasing efficiency in microsystems due to its basic design configuration. The inclusion of twisted helical improves fluid mixing by causing chaotic advection and increasing the surface area accessible for mass transfer. Overall a micromixer with T-junction and twisted helical insert can improve mixing and thermal efficiency by reducing entropy generation and optimizing synergy angle with velocity and temperature moving in the same direction. Overall, the entropy generation and synergy angle are significant characteristics to consider when building micromixers for thermal efficiency and miscible fluid mixing. Making the comparison between these configurations indicates that the highest mass mixing and heat mixing performance is obtained for θ=90° configuration.

## CRediT authorship contribution statement

**Abdelkader Mahammedi:** Writing – original draft, Methodology, Investigation, Formal analysis, Data curation, Conceptualization. **Naas Toufik Tayeb:** Writing – original draft, Visualization, Resources, Data curation. **Jin-Hyuk Kim:** Writing – review & editing, Supervision. **Shakhawat Hossain:** Writing – review & editing, Supervision.

## Declaration of competing interest

The authors declare that they have no known competing financial interests or personal relationships that could have appeared to influence the work reported in this paper.

## References

[1] Haghighinia A., Movahedirad S. (2019). Fluid micro-mixing in a passive microchannel: comparison of 2D and 3D numerical simulations. Int. J. Heat Mass Tran..

[2] Cai G., Xue L., Zhang H., Lin J. (2017). A review on micromixers. Micromachines.

[3] Kunti G., Bhattacharya A., Chakraborty S. (2017). Rapid mixing with high-through- put in a semi-active semi-passive micromixer. Electrophoresis.

[4] Mahammedi A., Ameur H., Ariss A. (2017). Numerical investigation of the performance of kenics static mixers for the agitation of shear thinning fluids. J. Appl. Fluid Mech..

[5] T. ManojDundi,V. Raju, V.P. Chandramohan, Characterization of mixing in an optimized designed T–T mixer with cylindrical elements. CJChE-01404;No of Pages 15.

[6] Kurnia J.C., Ahmadihosseini A., Sasmito A.P. (2022). Flow behavior and mixing of single-phase laminar Newtonian miscible fluid in T-junction micromixer with twisted mixing channel-A numerical study. Chemical Engineering and Processing-Process Intensification.

[7] Galletti C. (2019). Numerical investigation of flow regimes in T-shaped micromixers: Benchmark between finite volume and spectral element methods. Can. J. Chem. Eng..

[8] Lee C.Y., Lin C., Hung M.F., Ma R.H., Tsai C.H., Lin C.H., Fu L.M. (2006). Experimental and numerical investigation into mixing efficiency of micromixers with different geometric barriers. Mater. Sci. Forum.

[9] Mariotti A., Galletti C., Mauri R., Salvetti M.V., Brunazzi E. (2021). Effect of stratification on the mixing and reaction yield in a T-shaped micro-mixer. Physical Review Fluids.

[10] Engler M., Kockmann N., Kiefer T., Woias P. (2004). Numerical and experimental investigations onliquid mixing in static micromixers. Chem. Eng. J..

[11] Ali Abbas Munawwar, YanqinBai Mohammad Mehdi Rashidi, MubashirBhatti Muhammad (2016). Analysis of entropy generation in the flow of peristaltic nanofluids in channels with compliant walls. Entropy.

[12] Ali Chamkha, Ismael Muneer, Kasaeipoor Abbas, Armaghani Taher (2016). Entropy generation and natural convection of cuo-water nanofluid in c-shaped cavity under magnetic field. Entropy.

[13] Ali Abbas Munawwar, YanqinBai Mohammad Mehdi Rashidi, Bhatti Muhammad Mubashir (2016). Analysis of entropy generation in the flow of peristaltic nanofluids in channels with compliant walls. Entropy.

[14] Nasiri Mohammad, Mehdi Rashidi Mohammad, Lorenzini Giulio (2016). Effect of magnetic field on entropy generation in a microchannel heat sink with offset fan shaped. Entropy.

[15] Chen K. (2005). Second-law analysis and optimization of microchannel flows subjected to different thermal boundary conditions. Int. J. Energy Res..

[16] Hossain S., Kim K. (2015). Mixing analysis in a three-dimensional serpentine split-and recombine micro mixer. Chem. Eng. Res. Des..

[17] Hossain S., Kim K. (2016).

[18] Kim D.S., Lee S.H., Kwon T.H., Ahn C. (2019). Serpentine laminating micromixer combining splitting/recombination and advection. Roy. Soc. Chem..

[19] Xia H.M., Wan S.Y.M., Shu C., Chew Y.T. (2005). Chaotic micromixers using two-layer crossing channels to exhibit fast mixing at low Reynolds numbers. Roy. Soc. Chem..

[20] Kockmann N., Kiefer T., Engler M., Woias P. (2006). Convective mixing and chemical reactions in microchannels with high flow rates. Sensor. Actuator. B Chem..

[21] Kockmann N., Kiefer T., Engler M., Woias P. (2006). Silicon microstructures for high throughput mixing devices. Microfluid. Nanofluidics.

[22] Wong S.H., Ward M.C.L., Wharton C.W. (2004). Micro T-mixer as a rapid mixing micromixer. Sensor. Actuator. B Chem..

[23] Matsunaga T., Nishino K. (2014). Swirl-inducing inlet for passive micromixers. RSC Adv..

[24] Bothe D., Stemich C., Warnecke H.J. (2006). Fluid mixing in a T-shaped micro-mixer. Chem. Eng. Sci..

[25] Gobby D., Angeli P., Gavriilidis A. (2001). Mixing characteristics of T-type microfluidics mixers. J. Micromech. Microeng..

[26] Yang I.D., Chen Y.F., Tseng F.G., Hsu H.T., Chieng C.C. (2006). Surface tension driven and 3-D vortex enhanced rapid mixing microchamber, Micro electromechanical Systems. J. Health.com.

[27] Chen X., Li T., Li X. (2016). Numerical research on shape optimization of microchannels of passive micromixers. IEEE Sensor. J..

[28] Chen X., Li T. (2017). A novel passive micromixer designed by applying an optimization algorithm to the zigzag microchannel. Chem. Eng. J..

[29] Chen X., Zhao Z. (2017). Numerical investigation on layout optimization of obstacles in a three-dimensional passive micromixer. Anal. Chim. Acta.

[30] Bejan A. (1995).

[31] Bejan A. (1982).

[32] Bejan A. (1979). A study of entropy generation in fundamental convective heat transfer. ASME J. Heat Transfer.

[33] Naas Tayeb N.T., Amar K., Sofiane K., Lakhdar L., Yahia L. (2020). Thermal mixing performances of shear-thinning non-Newtonian fluids inside Two-Layer Crossing Channels Micromixer using entropy generation method: comparative study. Chemical Engineering and Processing-Process Intensification.

[34] Haddad O., Abuzaid M., Al-Nimr M. (2004). Entropy generation due to laminar incompressible forced convection flow through parallel-plates microchannel. Entropy.

[35] FatihSelimefendigil Hakan F. Öztop, Chamkha Ali J. (2016). MHD mixed convection and entropy generation of nanofluid filled lid driven cavity under the influence of inclined magnetic fields imposed to its upper and lower diagonal triangular domains. J. Magn. Magn Mater..

[36] Abbassi H. (2007). Entropy generation analysis in a uniformly heated microchannel heat sink. Energy.

[37] Ali Chamkha, Ismael Muneer, Kasaeipoor Abbas, Armaghani Taher (2016). Entropy generation and natural convection of cuo-water nanofluid in c-shaped cavity under magnetic field. Entropy.

[38] Erbay L.B., Yalcln M.M., Ercan M.S. (2007). Entropy generation in parallel plate microchannels. Heat Mass Tran..

[39] Avci M., Aydin O. (2007). Second law analysis of heat and fluid flow in microscale geometries. Int. J. Exergy.

[40] Sadeghi A., Asgarshamsi A., Saidi M.H. (2009, January).

[41] Jafari A., Ghazali N.M. (2010, June).

[42] Tu S.M., Torii S., Shih Y.C. (2011, January).

[43] Gillispie A.M., Lemley E.C. (2017, November).

[44] Yilmaz M. (2001). Performance evaluation criteria for heat exchangers based on second law analysis. Exergy.

[45] Al-Obaidi S. (2011).

[46] (2009). ANSYS FLUENT 12.0 Theory Guide.

[47] Shah I., Kim S.W., Kim K., Doh Y.H., Choi K.H. (2019). Experimental and numerical analysis of Y-shaped split and recombination micro-mixer with different mix- ing units. Chem. Eng. J..

[48] Douroum E., Laouedj S., Kouadri A., Naas T.T., Khelladi S., Benazza A. (2021). High hydrodynamic and thermal mixing performances of efficient chaotic micromixers: a comparative study. Chemical Engineering and Processing-Process Intensification.

[49] BaheriIslami S., Khezerloo M. (2017). Enhancement of mixing performance of non-Newtonian fluids using curving and grooving of microchannels. J. Appl. Fluid Mech..

[50] Gidde R.R., Pawar P.M. (2020). Flow feature and mixing performance analysis of RB-TSAR and EB-TSAR micromixers. Microsyst. Technol..

[51] Ko T.H. (2006). Numerical analysis of entropy generation and optimal Reynolds number for developing laminar forced convection in double-sine ducts with various aspect ratios. Int. J. Mass Heat Transfer.

[52] Kareem Akhtar (2023). Heat transfer augmentation and entropy generation analysis of microchannel heat sink (MCHS) with symmetrical ogive-shaped ribs. Energies.

